# Delivery of Islatravir via High Drug‐Load, Long‐acting Microarray Patches for the Prevention or Treatment of Human Immunodeficiency Virus

**DOI:** 10.1002/adhm.202403615

**Published:** 2025-01-22

**Authors:** Qonita Kurnia Anjani, Ashley R. Johnson, Akmal H. Sabri, Ryan Lutz, Steven Tignor, Jeanine Ballard, Nathan Rudd, Li Zhao, Lalitkumar K. Vora, Stephanie E. Barrett, Angela Wagner, Ryan F. Donnelly

**Affiliations:** ^1^ School of Pharmacy, Queen's University Belfast Medical Biology Centre 97 Lisburn Road Belfast BT9 7BL UK; ^2^ Sterile and Specialty Products Merck & Co., Inc. 2000 Galloping Hill Road Kenilworth NJ 07033 USA; ^3^ Absorption, Distribution, Metabolism & Excretion Merck & Co., Inc. 770 Sumneytown Pike West Point PA 19486 USA; ^4^ Small Molecule Analytical Research and Development Merck & Co., Inc. Rahway NJ 07065 USA; ^5^ Pharmaceutical Sciences R&D Merck Animal Health 126 E. Lincoln Avenue Rahway NJ 07065 USA

**Keywords:** HIV, islatravir, long‐acting, microarray patches, prevention, treatment

## Abstract

This research focuses on developing and characterizing islatravir‐loaded dissolving microarray patches (MAPs) to provide an effective, minimally invasive treatment option for human immunodeficiency virus (HIV‐1) prevention and treatment. The research involves manufacturing these MAPs using a double‐casting approach, and conducting in vitro and in vivo evaluations. Results show that the MAPs have excellent needle fidelity, structural integrity, and mechanical strength. in vitro studies demonstrate that the MAPs can penetrate skin up to 580 µm and dissolve within 2 hours. Permeation studies reveal that the delivery efficiency of islatravir across the skin is around 40%. In rodent models, these dissolving MAPs sustain islatravir delivery for up to 3 months. Scaling up the MAPs and increasing drug loading produced detectable levels in minipig. Projections from animal data suggest that these dissolving MAPs can achieve effective islatravir levels for a month after a single application in humans. These findings indicate dissolving MAPs as a minimally invasive approach to sustained release of islatravir.

## Graphical abstract

Islatravir‐loaded dissolving microarray patches (MAPs) provide a minimally invasive, long‐acting solution for HIV‐1 prevention and treatment. In rats, sustained islatravir delivery lasts up to three months, while in minipigs, efficacious plasma levels are detected for six days. Scaled‐up projections indicate monthly dosing in humans, showcasing the potential of MAPs for sustained antiretroviral therapy.

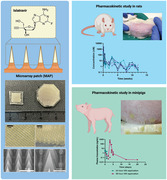

## 1 Introduction

The advent, improvement, and broader use of highly active retroviral therapy (HAART) since the mid‐1990s has transformed the lives of patients living with human immunodeficiency virus (HIV‐1) .^[^ ^1^ ^]^ While life expectancies of those who have achieved immune reconstitution and remain virologically suppressed are now close to the baseline,^[^ ^2^ ^]^ the next frontier of drug products for HIV‐1 treatment and prevention will seek to enhance the patient experience to improve adherence and restore a sense of normalcy in patients living with HIV‐1.^[^ ^3^, ^4^, ^5^ ^]^ To this end, some long‐acting injectables of antiretrovirals have been developed in order to reduce dosing frequencies, such as CABENUVA®, a once‐monthly combination of the integrase inhibitor cabotegravir and the non‐nucleoside reverse transcriptase inhibitor rilpivirine dosed as a crystalline suspension.^[^ ^6^ ^]^ SUNLENCA®, a long‐acting injectable formulation of the HIV‐1 capsid inhibitor lenacapavir has also been recently approved in Europe for once‐every‐six‐month administration.^[^ ^7^ ^]^

These approvals represent significant advances in the field, but challenges to successful implementation and patient acceptance still exist.^[^ ^5^, ^8^ ^]^ Viiv Healthcare's recent CUSTOMIZE study investigated barriers to implementation in US‐based clinics, including universities, private clinics, and healthcare maintenance organizations, among others.^[^ ^8^ ^]^ The factor identified as most interfering with patients’ ability to receive injections was injection pain or soreness.^[^ ^8^ ^]^ While acceptability was high across multiple types of clinics, some key operational changes were also instituted, including extending clinic hours, purchasing additional refrigerators, and implementing new tracking and reminder systems to enable monthly injections.^[^ ^8^ ^]^ Therefore, an approach that minimizes injection site pain and operational barriers to therapy would improve the patient experience and may increase the uptake of long‐acting drug products in the treatment and prevention of HIV‐1.

Microarray patches (MAPs) tested since the late 1990s have reduced or eliminated clinical injection site pain through the application of arrays of tiny needles that evade nerve endings buried deep in the skin. While such patches have not yet been marketed (largely due to challenges associated with manufacturing, quality systems, and regulatory processes), successful commercialization and uptake could eventually reduce the amount of healthcare worker intervention in the delivery of medications through self‐administration or administration in local pharmacies. In a global context, MAPs have the potential to enable delivery to regions with poor cold‐chain storage through room temperature stability.^[^ ^9^, ^10^ ^]^

For these reasons, several prior reports have investigated the delivery of antiretrovirals utilizing MAP technology.^[^ ^11^, ^12^, ^13^, ^14^, ^15^, ^16^, ^17^ ^]^ While cabotegravir and rilpivirine have also been successfully formulated and co‐administered in MAP, maximal drug loadings were ≈2.8 mg per 0.36 cm^2^ patch. In order to administer a dose consistent with once monthly CABENUVA (400–900 mg in total), a MAP size of ≈50–120 cm^2^ would be required.^[^ ^18^, ^19^ ^]^ Such impractically large MAP sizes are unlikely to be a preferred option for patients living with HIV as it will not only be uncomfortable to wear but would result in difficulty in patch application that could culminate in treatment failure. Therefore, there is an impetus to fabricate a novel MAP system capable of encapsulating a highly potent antiretroviral agent such as a nucleoside reverse transcriptase translocation inhibitor and delivering that agent at therapeutic levels from a reasonable patch size over extended therapeutic durations.

Islatravir is a desoxyadenosine analog being developed by Merck & Co., Inc., Rahway, NJ, USA for the treatment and prevention of HIV‐1 infection. Islatravir's novel structure inhibits reverse transcriptase via multiple mechanisms, resulting in high potency and a high barrier to resistance. Islatravir's combination of high potency and slow human clearance makes it a promising low‐dose, long‐acting medication.^[^ ^20^ ^]^ In a phase 1 study, islatravir implant doses as low as 62 mg produced islatravir triphosphate levels exceeding the efficacy target for more than 12 weeks, exemplifying its utility as a long‐acting medication.^[^ ^21^ ^]^ Additionally, Pons‐Faudoa et al. reported that subcutaneous long‐acting nanofluidic implants effectively controlled islatravir release and reduced viral load in Simian immunodeficiency virus‐infected macaques.^[^ ^22^ ^]^ Kim et al. further supported subcutaneous implants, modeling the pharmacokinetics, pharmacodynamics, toxicity, and prophylactic efficacy.^[^ ^23^ ^]^ However, subcutaneous implants require surgical procedures for administration, potentially limiting their accessibility. In contrast, MAPs are minimally invasive, penetrating the *stratum corneum* without reaching dermal nerves or blood vessels, thus improving patient compliance and adherence. While Kinvig et al. proposed MAP‐mediated islatravir delivery using physiologically based pharmacokinetic modeling,^[^ ^12^ ^]^ formulation and in vivo delivery of islatravir via MAPs have not been previously reported. This study presents, for the first time, successful formulation and long‐acting delivery of islatravir using MAPs in rodents and minipigs. Biopharmaceutic modeling suggests the potential for multi‐month therapeutic delivery with MAPs of approximately 20 cm^2^ in size.

## 2 Results

### 2.1 MAP Characterization Studies

**Figure 1:**
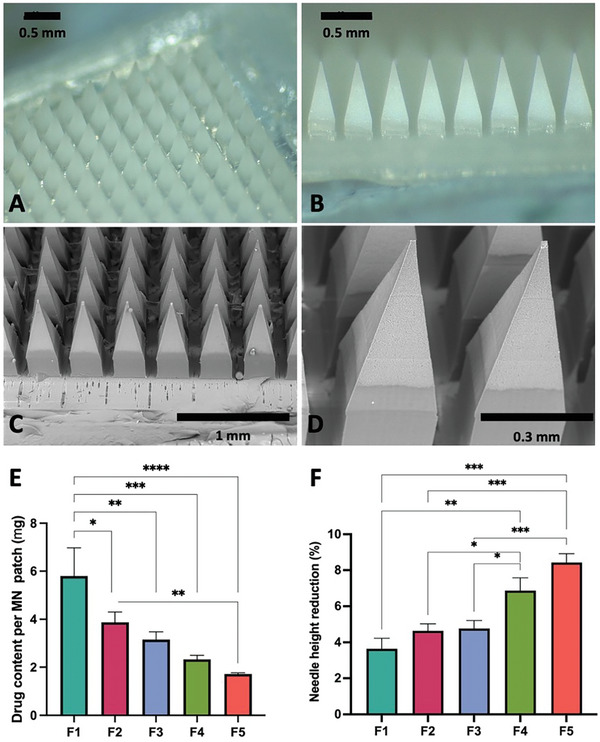
A,B) Digital and C,D) SEM images of representative islatravir‐loaded MAPs. E) Drug loading per MAP for formulations F1, F2, F3, F4, and F5 (means + SD, n = 3). F) Percentage of needle height reduction following application of a force of 32 N (mean + SD, n = 20).

In the current work, a series of dissolving MAPs loaded with islatravir were fabricated via a two‐step casting approach. Images of representative patches of F1 are shown in **Figure** **1** (alternate formulations are shown in the SI). The opaque drug‐containing layer was localized to the pyramidal section of the obelisk microprojections as intended, while the baseplate remained flat and clean. As presented in Table S3 (Supporting Information), the drug loading and polymer components varied across formulations, with F1 containing the highest theoretical solid content in the needle tips (45.1%) and F5 the lowest (18%). Although all formulations displayed homogeneous and sharp tips, decreasing drug and polymer content in the tips resulted in reduced mechanical robustness. The higher polymer content in F1 functioned as a binder, helping to interlock drug particles within the needle layer and reducing the formation of highly porous needle structures post‐drying.^[^ ^24^, ^25^ ^]^ This phenomenon appears to depend significantly on the payload incorporated in the dissolving MAP.

MAP drug loading decreased progressively from formulation F1 to F5 (Figure 1E), consistent with the reduction in islatravir cast into the needle tips of the polydimethylsiloxane molds, as expected. A maximum total drug load of ≈5.8 mg was encapsulated into a 16 × 16 MAP patch for formulation F1, with an overall patch size of 0.36 cm^2^.

The mechanical resistance of the formulations to axial compressive force was also investigated. In this test, a 32 N compressive force (analogous to the force exerted by manual thumb pressure during skin application) was applied to the patches for 30 seconds. The measured percent height reduction was less than 10% for all formulations studied (Figure 1F), confirming sufficient mechanical robustness to enable skin insertion.^[^ ^26^, ^27^ ^]^ The percent reduction in height decreased with reduced water content in the initial casting film. Formulation F1 exhibited the greatest mechanical robustness, attributed to its lower presumed porosity within the microneedle tips due to higher polymer and drug content.

### 2.2 Ex Vivo Porcine Skin Penetration and Dissolution Study

**Figure 2:**
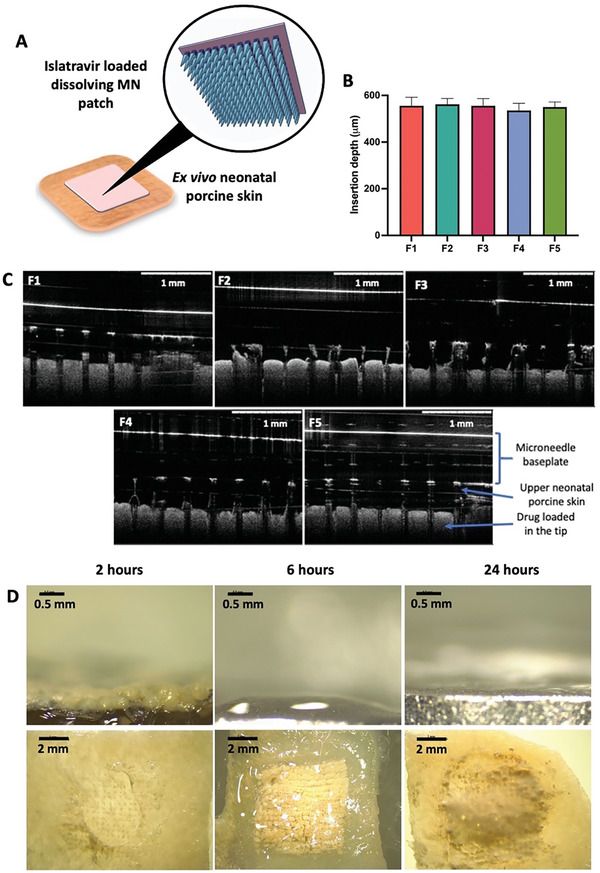
A) Insertion and dissolution of islatravir‐loaded MAPs in ex vivo neonatal porcine skin. B) Insertion depth of MAPs into ex vivo neonatal porcine skin as quantified from OCT images (means + SD., n = 20). C) OCT images of respective islatravir‐loaded MAP formulations into ex vivo neonatal porcine skin. D) Images of islatravir‐loaded MAPs (F1) dissolving in the ex vivo neonatal porcine skin at 2, 6, and 24 hours acquired by optical microscopy.

We further evaluated the overall microneedle insertion depth following ex vivo skin application to porcine tissue as shown in **Figure** **2**. In situ visualization using optical coherence tomography (OCT) indicated that the drug‐loaded layer of the needle tips was fully embedded into the skin with an insertion depth of ≈550 µm. This would suggest that when the patches are applied to the skin, the base layer should dissolve and embed the drug‐containing layer into the skin, forming micro‐depots for sustained drug release. Although the drug loading/porosity of the formulation had a significant impact on the overall mechanical properties of the needles, this parameter did not have a statistically significant (*p* > 0.05) effect on the insertion depth of microneedles into the skin, with all formulations inserting to a depth of ≈550 µm.

In addition to the insertion of MAPs into the skin, we also evaluated the time required for the needles to dissolve into the skin following application (Figure 2D). We observed that all of the needle layers of the patches have fully dissolved and detached from the baseplate following 2 hours of skin application. This resulted in the formation of off‐white miniature depots within the skin observed under the stereo microscope. When the formulations were removed from the skin after 2 and 4 hours of skin application, we observed that only the polymeric baseplate, fabricated from PVP and glycerol, remained on the patch post‐removal. After 24 hours, the baseplates of these formulations fully dissolved, forming a polymeric gel layer on top of the skin. The dissolution kinetics of all five MAP formulations were qualitatively observed similar.

### 2.3 Ex Vivo Delivery Efficiency in Porcine Skin

**Figure 3:**
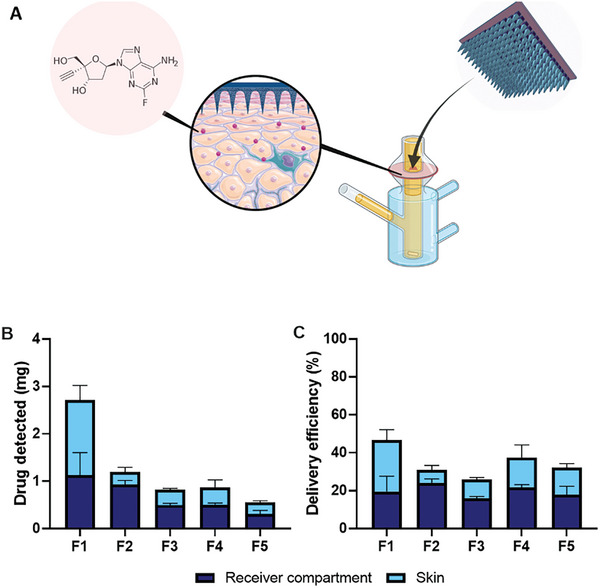
A) Schematic illustrating the Franz cell setup to investigate the permeation profile of islatravir‐loaded dissolving MAPs. B) Total delivery and distribution of islatravir following application of MAPs from respective formulations. C) Delivery efficiency of islatravir into receiver compartment and neonatal porcine skin following MAP application (means + SD, n = 3).

A skin deposition study utilizing a Franz cell setup was employed to quantify MAP mediated delivery of islatravir into and across the skin over a 24‐hours period (**Figure** **3**). Formulation F1, which has the highest drug loading out of the series of dissolving MAPs also resulted in the highest amount of islatravir administered into and across the skin with up to 2.7 mg of the drug being delivered (Figure 3B). In contrast, F2‐F4 resulted in ≈0.8–1.0 mg of islatravir being delivered into and across the skin. Lastly, F5 which has the lowest drug loading had the lowest amount of drug being delivered with only 0.5 mg of the compound being delivered. With regards to delivery efficiency, we observed that formulation F1, which had the highest drug loading and amount of drug delivered also exhibited the highest delivery efficiency at 46.7% (Figure 3C). Nevertheless, formulation F2‐F4 exhibited a delivery efficiency of ≈25–37%. This suggests that higher drug loading enhances delivery, as more drug deposited in the skin is gradually released over time. As shown in Figure 3B, drug permeation into the receiver compartment ranged from approximately 0.3 to 1 mg. This is likely due to the concentration gradient principle, where higher drug concentrations in the skin promote migration to the lower‐concentration receiver compartment. Thus, increased drug loading in the patch results in greater overall delivery. For all formulations, delivery efficiencies were relatively similar and not statistically different (*p* > 0.05). Based on these in vitro delivery results and drug loading data, formulation F1 was selected for further in vivo evaluation and stability testing.

### 2.4 Stability Testing

The stability of each MAP formulation was evaluated over a 3‐month period at a number of different conditions (Table S1), ranging from refrigerated in pouch under ambient humidity through 40°C and 75% relative humidity out of pouch. The chemical and physical stability of islatravir as well as the mechanical stability of the MAPs were evaluated under all conditions. No changes in the chemical composition/impurity profile of the drug were observed over the three‐month study (Table S2, Supporting Information). No changes in the X‐ray diffraction pattern of the drug were observed, indicating that the drug remained crystalline throughout the study (Figure S3, Supporting Information).

**Figure 4:**
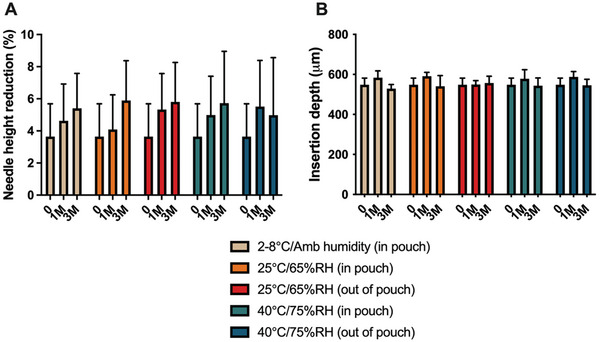
A) Percentage of needle height reduction and B) insertion depth of MAPs into ex vivo neonatal porcine skin after storage at different conditions (means + SD, n = 20). M = month.

The mechanical stability of the MAPs was also evaluated after exposure to all conditions, both by measuring the amount of deformation after application of a 32 N force and by measuring microneedle insertion depth upon thumb insertion into porcine skin ex vivo (**Figure** **4**). It can be seen that regardless of the storage condition and duration, all the MAPs displayed no significant changes (*p* > 0.05) in needle height reduction. All the MAPs displayed an average height reduction between 4% and 6%. Previous studies on dissolving MAPs have indicated that MAPs displaying a height reduction of less than 10% would possess sufficient mechanical robustness to pierce and puncture the skin upon application.^[^ ^28^, ^29^ ^]^ This was further validated by a skin insertion study (Figure 4B) that showed all the MAPs, regardless of the storage condition and duration, were able to puncture the skin as visualized via OCT imaging to an insertion depth of ≈580 µm. This is the ideal intradermal depth needed for the formulation to be deposited in order for the drug to dissolve from the depot into the dermal microcapillaries before reaching the systemic circulation.^[^ ^30^, ^31^ ^]^

Interestingly, despite being fabricated from hydrophilic and hygroscopic polymers such as poly(vinyl alcohol) (PVA), poly(vinyl pyrrolidone) (PVP), and glycerol, the mechanical strength and insertion capabilities of the MAPs were not compromised, even under high‐temperature and high‐humidity conditions. This resilience can be attributed to the use of a unique 3D‐printed storage box for dissolving MAPs, as previously reported.^[^ ^28^ ^]^ This box effectively maintained MAP stability for 30 days under varying temperature and humidity conditions.^[^ ^28^ ^]^ In this study, the box was modified by placing it inside a heat‐ and humidity‐resistant aluminum pouch, providing additional protection against direct exposure to heat and moisture. This combined packaging solution—consisting of the MAP‐box and aluminium pouch—offers a practical option for the storage and transportation of dissolving MAPs. Such a packaging design could potentially be adopted for clinical use, facilitating the transition of MAPs into commercially available pharmaceutical products.

### 2.5 Pharmacokinetic Study in Rats

**Figure 5:**
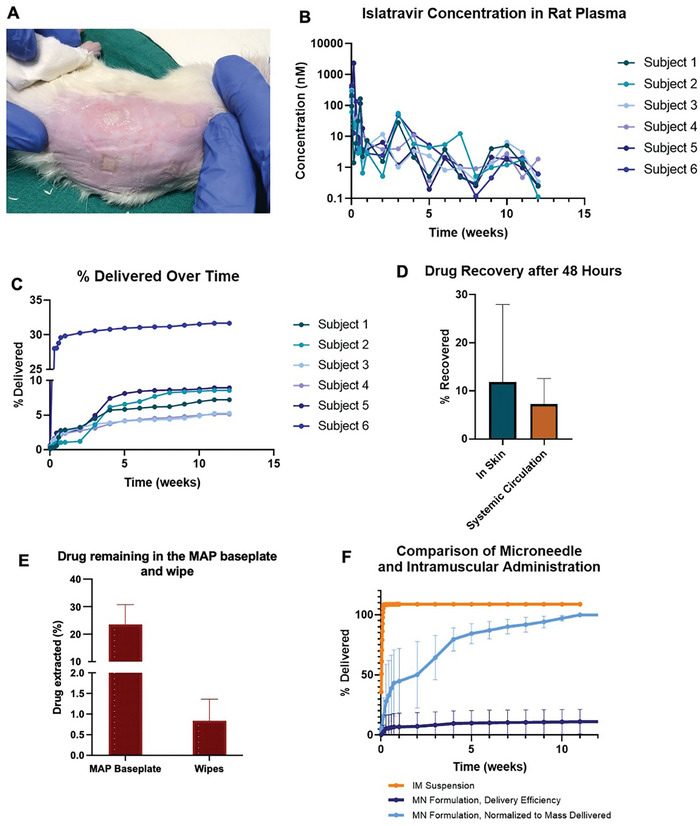
Rat pharmacokinetics after MAP and intramuscular administration. A) MAPs after being removed from the rats after 24‐hour application. B) Islatravir concentration in rat plasma after MAP administration. C) Percentage of total drug administered to the skin in systemic administration over the study period. D) Amount of drug recovered from the skin and systemic circulation 48 hours after MAP administration (means + SD., n = 6). E) Comparison of delivery efficiency and kinetics resulting from administration of MAP and an intramuscular suspension (means ± SD., n = 6).

In order to assess pharmacokinetics resulting from MAP administration, four MAPs were applied to Sprague–Dawley rats for a period of 24 hours; patches were then removed and islatravir levels in plasma were monitored over a 3‐month period. As shown in **Figure** **5**A, the MAPs fully dissolved, and islatravir‐loaded tips were embedded into the skin, indicated by white spots. Upon removing the Microfoam adhesive frames, Tegaderm film, and kinesiology tape, no skin irritation or inflammation was observed. Figure 5B shows that islatravir remained detectable in plasma throughout the three‐month study in all six animals. Early timepoints were characterized by a degree of burst release over the first week followed by a comparably more consistent plasma levels over the remainder of the study. An analysis of release rate from the patches (Figure S4, Supporting Information) shows that pseudo‐steady state release rates were approximately 10 µg d^−1^. Of note, release rates from the MAPs closely mirror the resulting plasma concentrations, indicating that the plasma concentrations are primarily dictated by the absorption phase, as expected given the rapid clearance of islatravir in rats. Subject‐to‐subject variability in plasma levels was high, with differences in plasma concentration between subjects measuring up to approximately one order of magnitude.

Over the three‐month study, only 11.1% ± 10.2% of the drug in the patch was cumulatively detected. Subject 6 exhibited significantly higher initial release, with over 30% of the administered drug detected in plasma (Figure 5C). The relatively low drug detection across subjects suggests two potential scenarios, 1) ≈10% of the drug loaded in the patches reaches plasma/skin, or 2) additional drug remains in the skin, expected to release over longer durations. To distinguish between these scenarios, an additional study was conducted in which rats were sacrificed 48 hours after MAP application (with a consistent 24‐hour wear period), and the total drug in plasma and skin was quantified (Figure 5D). On average, 7.5% ± 5.4% of the total drug was detected in plasma, and 11.8% ± 16.1% was isolated from the skin, indicating a total drug delivery efficiency of 19.0% ± 14.3%. Most of the delivered drug was detected within the study period, suggesting a relatively low overall efficiency with minimal additional drug expected to circulate at later time points.

To calculate the mass balance, the drug remaining in the MAP baseplate and in tissue paper used to clean the sticky residue from rat skin after patch removal was quantified. As shown in Figure 5E, 23.52% ± 7.21% of the total drug remained in the undissolved baseplate, and 0.84% ± 0.52% was extracted from the cleaning tissue. This indicates a total drug recovery of ≈44% (considering delivered and residual amounts). The unaccounted drug may be attributed to skin enzymatic metabolism or systemic metabolism.^[^ ^32^, ^33^ ^]^ Since this is the first study using islatravir delivered transdermally, future studies should investigate potential skin enzymatic activity affecting drug metabolism.

In comparison, pharmacokinetic analysis of intramuscular injection of an islatravir suspension showed significantly higher drug delivery efficiency (Figure 5F), with 100% of the dosed drug detected in plasma within 24 hours. Conversely, the MAP formulation demonstrated more sustained release over the 3‐month period (Figure 5F), with a notable decrease in dose‐normalized C_max_ from 0.157 mg^−1^ L^−1^ mg^−1^ dose for intramuscular suspension to 0.015 mg L^−1^ mg^−1^ dose for the MAP formulation (Figure S4, Supporting Information). This sustained release supports the hypothesis that microneedle tips successfully deposit the drug into the skin, where it gradually dissolves over time (Figure 5A).

### 2.6 Fabrication and Characterization of Larger MAPs

**Figure 6:**
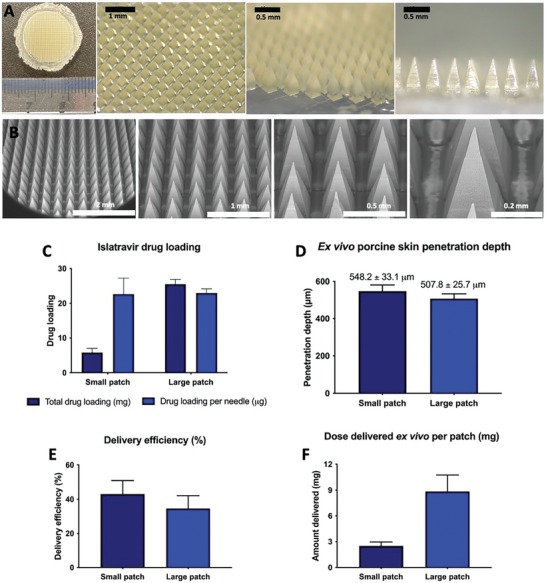
A) Digital and B) SEM images of islatravir‐loaded large MAPs, formulation F1. C) Islatravir drug loading in large and small patches, represented as both the total drug load in each batch and the amount of islatravir per needle (means + SD, n = 6). D) Insertion depth of large MAPs into ex vivo neonatal porcine skin as quantified from OCT images (means + SD, n = 20). E) Delivery efficiency and F) total delivery of islatravir into receiver compartment and neonatal porcine skin following application of small and larger MAP application (means + SD, n = 4).

In the previous section, we demonstrated that islatravir can be successfully fabricated into a postage stamp‐sized dissolving MAP (0.36 cm^2^), capable of delivering the compound transdermally in rats for at least 12 weeks. To support large animal studies, the islatravir‐loaded dissolving MAPs were scaled up from a 0.36 cm^2^ patch containing 256 microneedles to a 1.76 cm^2^ patch containing 1110 microneedles, increasing the number of microneedles approximately fourfold and the patch area 4.5‐fold. Images of the larger formulation F1 MAPs in **Figure** **6**A,B show that the microneedles retain the same geometry and integrity as those on the smaller patches. The large patch in the present study was designed to feature a high needle density and excellent skin insertion efficiency, achieved through precise geometric configuration. The baseplate of each MAP spans a total surface area of 1.76 cm^2^ and includes 1110 extended pedestal microneedles. Each microneedle consists of a cuboidal base (300 µm in both height and width) and a pyramidal tip (600 µm in height) with a 10 µm tip radius to aid skin penetration. The needles are arranged with a 100 µm spacing at the baseplate. This configuration provides a dense array (1110 needles per 1.76 cm^2^) to enhance drug loading capacity and penetration depth while mitigating the “bed of nails effect”.^[^ ^34^ ^]^ The pyramidal tips, with a 2:1 length‐to‐width aspect ratio, are designed to deliver sufficient mechanical strength and enable effective insertion into the skin.^[^ ^34^, ^35^, ^36^ ^]^ Moreover, the geometry allows for efficient demolding without needle breakage,^[^ ^34^, ^35^, ^36^ ^]^ facilitating large‐scale production of intact MAPs.

Scaling up the array size increased drug loading from 5.8 mg to 25.5 mg, with the islatravir mass per needle remaining constant (Figure 6C). Mechanical characterization showed less than a 10% reduction in height after applying 32 N of force, suggesting that scaling up had minimal impact on the formulation's mechanical properties (Figure S5, Supporting Information). MAP application and OCT imaging in ex vivo porcine skin confirmed that the drug‐containing needle layer was successfully inserted into the skin (Figure S5, Supporting Information), with an approximate insertion depth of 500 µm (Figure 6D), slightly less than the 550 µm penetration depth observed with smaller patches. These differences are expected, as the application force is distributed over a larger number of needles in the larger patch. Nevertheless, both patch sizes effectively inserted the drug‐containing tips into the dermal layer. The large MAP delivered 8.8 mg of islatravir into the skin, achieving a delivery efficiency of 34.5%, as measured using the Franz diffusion cell apparatus. While delivery efficiency was slightly lower than with the smaller patches, as expected due to the reduced penetration depth, the total amount delivered increased ≈2.5‐fold, enabling progression to minipig studies with the larger patch sizes.

### 2.7 Pharmacokinetic Study in Minipigs

**Figure 7:**
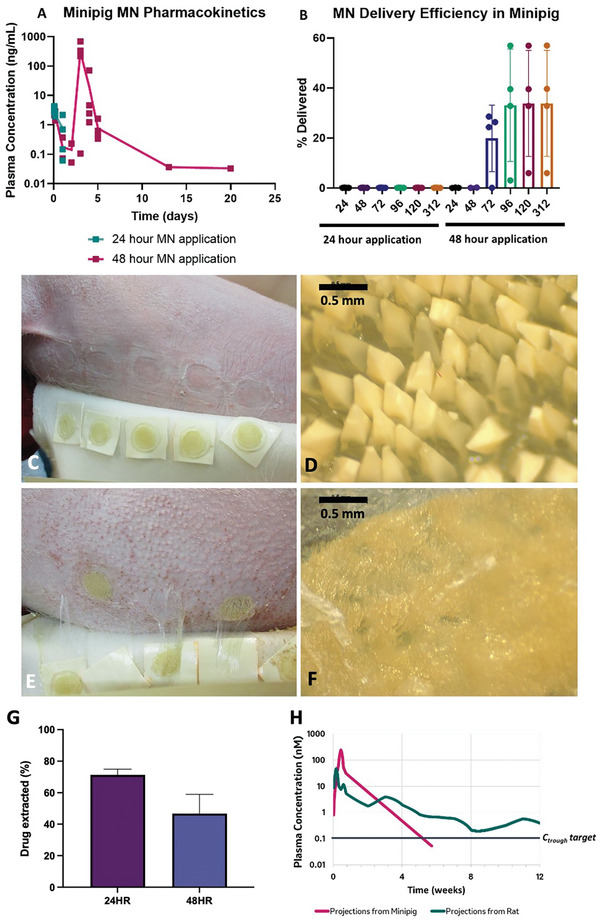
Minipig pharmacokinetics after MAP administration. A) Plasma concentration over time and B) delivery efficiency after a 24‐hour and a 48‐hour application period (means ± SD, n = 4). C) MAPs after being removed from the minipig after 24 hours. D) The appearance of the needles after removal at the 24‐hour mark. E) MAPs after being removed from the minipig after 48 hours. F) The appearance of the needles after removal at the 48‐hour mark. G) The amount of drug extracted from the remaining baseplate after 24 and 48 hours (means ± SD, n = 20). H) Human dose projections.

Islatravir MAPs were further evaluated in minipigs to gather extensive data and provide insights into their potential human performance. Minipigs are widely used in transdermal drug delivery studies due to their skin's similarity to human skin in thickness and composition.^[^ ^37^, ^38^, ^39^, ^40^ ^]^ In this study, ten large MAPs (with a total of 11,100 microneedles) were applied to each minipig. In one study arm, patches were worn for 24 hours before removal. During this period, islatravir was detectable in plasma, but no additional drug was detected post‐removal (**Figure** **7**A). After 24 hours, ≈80% of the drug remained in the MAPs (Figure 7G), and the microneedles were not fully dissolved (Figure 7D).

In a second arm of the study, extending the wear time to 48 hours resulted in detectable plasma levels of islatravir for at least 5 days, with one minipig maintaining levels for up to 20 days (Figure 7A). Delivery efficiency increased significantly from less than 1% with 24‐hour patches to 6–57% with 48‐hour applications (Figure 7B). Most microneedles dissolved after 48 hours (Figure 7F), leaving ≈42% of islatravir in the baseplate (Figure 7G).

Islatravir delivery in this study likely involved two mechanisms. First, the embedded portion of the microneedles directly delivered the drug into the skin. Second, interstitial fluid interacting with the unembedded portion facilitated gradual microneedle dissolution, enabling sustained drug diffusion into the skin. The concentration gradient played a critical role in this process, as drug outside the skin relied on this gradient for passive diffusion.^[^ ^41^ ^]^ Optimizing microneedle design to enhance penetration depth could improve delivery efficiency and reduce reliance on unembedded portions.

The microneedle tips required at least 48 hours to fully dissolve in minipigs. Formulated with a hydrosoluble matrix of PVA and PVP (7:1 islatravir‐to‐polymer ratio), the MAP tips dissolved slowly, likely due to islatravir's low solubility (less than 1 mg mL^−1^ in water). As shown in Figures 5A,E, embedded MAP tips gradually released islatravir into deeper skin layers, aided by the compound's hydrophobic nature. The absence of eccrine sweat glands in minipig skin, unlike human skin, may have further slowed dissolution due to reduced sweat secretion under occlusion.^[^ ^42^, ^43^ ^]^

Previous studies with hydrogel‐forming MAPs in minipigs demonstrated controlled drug release over five days,^[^ ^39^ ^]^ but optimal application times for dissolving MAPs in minipigs remain underexplored.^[^ ^44^, ^45^, ^46^ ^]^ The observed increase in drug delivery efficiency after 72 hours suggests that extended contact time between the microneedles and interstitial fluid plays a critical role in enhancing dissolution and diffusion then systemic release. During the first 24 hours, the drug embedded in the microneedle tips was less accessible to interstitial fluid, explaining the lower drug release in this period. Between 24 and 48 hours, prolonged contact allowed the microneedles to fully dissolve, facilitating drug release into systemic circulation and resulting in the observed increase in delivery. This extended dissolution time aligns with the gradual plasma release profile and explains the significant increase in delivery efficiency between 24 and 48 hours. Furthermore, a 48‐hour wear time is practical and shorter than conventional transdermal patches like Butrans and Transtec, which require 7‐day and 4‐day applications, respectively.^[^ ^47^ ^]^

### 2.8 Modeling Human Dose Projection and Patch Size

The potential of islatravir as a long‐acting medication is associated with its extended clearance in humans.^[^ ^48^ ^]^ Therefore, in order to predict achievable treatment durations resulting from MAP administration, dose‐normalized release rates resulting from administration of MAPs in rats and in minipigs were convolved with modeled islatravir intravenous pharmacokinetic in humans. In order to calculate possible plasma levels after dosing a 20 cm^2^ MAP, dose‐normalized projected human plasma projections were multiplied by a 322 mg dose, which is reflective of the total amount of drug that could be encapsulated in a 20 cm^2^ MAP assuming dose increases proportionally to patch surface area. Projected plasma concentrations were compared against a 0.1 nm plasma concentration target, which has previously been demonstrated to be consistent with greater than a one log reduction in plasma HIV1‐RNA (Figure 7H). Projections from rat were demonstrated to be consistent with meeting the target for over three months, whereas projections from minipig were consistent with at least a one month duration.

## 3 Discussion

This article presents the first reported use of MAPs for long‐acting delivery of islatravir, marking a significant step toward delivering deoxyadenosine analogs using next‐generation, minimally invasive technologies aimed at improving patient adherence. Dissolvable islatravir MAPs were formulated at up to 87% total solids loading and successfully penetrated skin, as evidenced by ex vivo porcine tissue testing and in vivo studies in rats and minipigs. Unlike prior attempts with less potent antiretrovirals, this effort demonstrated proof of concept for extended delivery (e.g., one month or longer) at therapeutic levels within a reasonable patch size of 20 cm^2^, approximately the size of three American quarters. The MAPs exhibited stability consistent with storage at 25°C and 40°C, addressing challenges associated with cold‐chain requirements, which often limit access in low‐resource settings. These findings highlight the potential of long‐acting MAP technology in HIV‐1 treatment and prevention, offering the prospect of improving patient adherence, increasing interest in HIV‐1 prevention regimens, and expanding access in lower‐income countries where refrigeration and medical personnel are limited.

While the presented work represents an exciting step, significant additional research is needed. High doses of islatravir in clinical studies have been associated with decreases in lymphocyte and CD4+ T‐cell counts, and current trials are focusing on lower doses anticipated to avoid these effects. Variability in delivery efficiency and plasma levels also necessitates further study. Drug release from MAP formulations involves three critical steps, 1) skin penetration, 2) microneedle‐tip detachment, and 3) tip dissolution, all essential for drug uptake. ex vivo studies demonstrated penetration of islatravir‐loaded MAPs to a depth of ≈550 µm, matching the drug‐loaded layer height. However, additional in vivo studies in minipigs, such as those employing optical coherence tomography, applicators, or varying tip heights, are needed to confirm sufficient penetration depth for consistent delivery.

Results from minipig studies underscore the importance of wear time in delivery efficiency. Rats, with thinner, more permeable skin, showed rapid microneedle dissolution within 24 hours, while minipigs required at least 48 hours due to their thicker, human‐like skin.^[^ ^49^, ^50^ ^]^ The relative drug levels in systemic circulation were lower and detected for shorter durations in minipigs due to their larger body mass and slower absorption. Burst release observed in minipigs after patch removal at 48 hours highlights the slower dissolution in thicker skin, necessitating extended application for optimal release. Improved delivery efficiency with longer wear time likely results from enhanced microneedle‐tip dissolution promoted by skin hydration. Future studies exploring MAP tip and backing compositions to modulate dissolution rates, as well as evaluating prolonged wear times, could refine design parameters for efficient drug absorption. Moreover, discrepancies in dose projections from rats and minipigs are largely attributed to species differences in metabolic rates and drug clearance. For instance, rats and minipigs exhibit faster hepatic metabolism and clearance than humans due to their higher metabolic rates and different enzyme profiles.^[^ ^51^, ^52^ ^]^ To enhance pharmacokinetic confidence, higher doses in minipigs should be investigated to extend detectable plasma drug levels.

The 20 cm^2^ patch design aligns with preclinical models predicting one to three months of therapeutic coverage with a 322 mg dose, consistent with previous findings despite differences in biopharmaceutical modeling.^[^ ^53^ ^]^ Large patch application is feasible; prior research showed no significant difference in insertion depth between large and single‐unit MAPs (0.5 cm^2^).^[^ ^53^ ^]^ Additionally, a 20 cm^2^ patch is comparable to marketed transdermal products like Nicotinell® (30 cm^2^) and Duragesic® CII (32–42 cm^2^),^[^ ^54^, ^55^ ^]^ making it practical and appropriate for islatravir delivery.

## 4 Conclusion

In conclusion, this study highlights the development and characterization of islatravir‐loaded dissolving MAPs with acceptable delivery efficiency that can administer the drug in a minimally invasive yet sustained fashion over several weeks as a potential formulation for the prevention and treatment of HIV. The results showed that the dissolving MAPs would be capable of releasing efficacious level of islatravir over the course of one month following a single application based on projections from minipig data. Consequently, it could be postulated that dissolving MAPs may offer a simple yet effective alternative treatment strategy to implants, albeit a shorter release period, in order to attain sustained release of islatravir. The ability to apply a MAP with a size of 20 cm^2^ on a monthly basis could simplify treatment for HIV patients thus obviating the need to adhere to complex and strict antiretroviral therapies.

## 5 Experimental Section

### MAP Formulation and Drug Encapsulation

Islatravir‐loaded MAPs were prepared using a double‐casting method. The first layer, consisting of the needle tips, was created by blending pure islatravir with an aqueous solution of PVA and PVP. Five different formulations were tested, as outlined in Table S3 (Supporting Information). To prepare the drug‐polymer mixture, islatravir was combined with the PVA‐PVP solution and mixed at 3000 rpm for 3 min using a SpeedMixer DAC 150.1 FVZ‐K (GermanEngineering, Hauschild & Co. KG, Hamm, Germany). After the initial mixing, deionized water was added to reduce the gel viscosity, followed by another mixing cycle at the same speed and duration. The resulting polymer–drug blend was cast onto a silicone mold with 16×16 microneedles (obelisk shape, 850 µm height, 300 µm base width, 300 µm interspacing, 0.36 cm^2^ patch area). The mold was then subjected to a positive pressure of 5 bar for 3 min (Protima, TÜV Rheinland, Cologne, Germany) to ensure the cavities were filled. Excess material was carefully removed from the surface using a clean spatula. The molds were placed back under 5 bar pressure for an additional 30 minutes. A silicone elastomer ring was affixed to the mold's surface using a 40% w/w PVA solution (9–10 kDa) as a temporary adhesive, and the formulation was left to air dry at room temperature for 24 hours. Once the system was fully dried, 850 µL of baseplate formulation (as per Table S4, Supporting Information) was applied on top of the needle layer, followed by centrifugation at 3500 rpm for 15 minutes. Finally, the molds were placed on a level surface and dried at 37.5°C for an additional 24 hours to ensure complete drying.

### MAP Visualization and Quantification of Drug Loaded in MAPs

The morphology of islatravir‐loaded MAPs was observed using a stereo microscope (Leica EZ4 D, Leica Microsystems, Milton Keynes, UK) and a SEM (Tabletop Microscope TM3030, Hitachi, Krefeld, Germany). SEM images were captured at a voltage of 15 kV under vacuum conditions. To determine the amount of islatravir encapsulated in the MAPs, each patch was placed in 5 mL of deionized water and sonicated for 3 hours to dissolve the polymer completely. Afterward, 5 mL of methanol was added to the solution, and it was sonicated for an additional 3 hours to ensure complete dissolution of the drug. The resulting mixture was diluted accordingly before being analyzed by high‐performance liquid chromatography (HPLC).

### Ex Vivo Skin Penetration Study and Mechanical Testing

Full‐thickness neonatal porcine skin was obtained from stillborn piglets within 24 hours of death and stored at −20°C until used. Prior to the study, the skin was thawed and equilibrated at room temperature by immersion in PBS (pH 7.4). Islatravir‐loaded MAPs were inserted into the full‐thickness skin using thumb pressure applied to the patch. A light microscope was used to visualize microneedle penetration. Needle insertion and dissolution in the skin were monitored with an EX‐101 OCT microscope (Michelson Diagnostics Ltd., Kent, UK), and images were analyzed using ImageJ software (NIH, Bethesda, MD, USA) to measure microneedle insertion length. For mechanical testing, the MAPs were evaluated using a TA‐TX2 Texture Analyser (Stable Microsystems, Haslemere, UK).^[^ ^56^, ^57^ ^]^ MAPs were compressed against a flat aluminum surface at a test speed of 0.5 mm s^−1^, with a force of 32 N applied for 30 seconds. The height of the microneedles was measured before and after compression, and the percentage reduction in needle height was calculated.

### Ex Vivo Skin Dissolution and Delivery Efficiency

A skin dissolution study was conducted to assess the dissolution timeline after inserting MAPs into ex vivo neonatal porcine skin. The skin, sourced from stillborn piglets within 24 h post‐mortem, was stored at −20°C until use. Before the experiment, full‐thickness neonatal porcine skin was equilibrated by immersing it in PBS (pH 7.4) for 30 minutes at 37°C. The MAPs were manually applied to the skin with thumb pressure for 30 seconds. To prevent dislodging, a cylindrical stainless steel weight of around 15 g was placed on top of the MAP. The samples were incubated in a thermostatically controlled chamber (Genlab incubator, Genlab Ltd., Cheshire, UK) set to 37°C and examined at 2, 6, and 24 hours by carefully removing the MAPs. Afterward, both the MAPs and skin were observed under a stereo microscope. Drug deposition from the formulations was evaluated using a Franz cell system (PermeGear, Inc., Hellertown, PA, USA). For each MAP formulation listed in Table S3 (Supporting Information), full‐thickness neonatal porcine skin was trimmed to fit the donor compartment of the Franz cells and adhered using cyanoacrylate glue (Stick it super glue, PLDZ Pattison House, Dublin, ROI) with the subcutaneous side facing the receiver chamber. MAPs were inserted into the *stratum corneum* side of the skin using manual thumb pressure for 30 seconds. The receiver chamber was filled with PBS (pH 7.4) and maintained at 37°C with a water jacket, stirred at 600 rpm. After 24 hours, the skin was removed from the Franz cell, homogenized in a 2 mL Eppendorf tube containing two stainless steel beads and 0.5 mL deionized water, using a Tissue Lyser LT (Qiagen Ltd., Manchester, UK) at 50 Hz for 15 minutes. An additional 1 mL of methanol was then added, and the homogenization process repeated. The skin and receiver compartment samples were centrifuged at 15,300 rpm for 15 minutes, and the supernatant was collected for HPLC analysis.

Islatravir quantification was performed using a reversed‐phase Agilent HPLC system (Agilent Technologies 1220 Infinity compact LC series, Agilent Technologies UK Ltd, Stockport, UK) with a UV detector. Separation was achieved on an XBridge C18 column (3.5 µm particle size, 4.6 mm internal diameter, 150 mm length, Waters, Dublin, Ireland). The samples were eluted using a mobile phase of 0.1% v/v phosphoric acid and acetonitrile (detailed in Table S5, Supporting Information) at a flow rate of 1 mL min^−1^. The analysis was conducted at 30°C with a 20 µL injection volume, and the run time was 10 minutes.

### Stability Testing

The stability of the islatravir‐loaded MAPs was assessed in terms of needle height reduction, ex vivo skin insertion, crystallinity, and drug content over time, with storage conditions detailed in Table S2 (Supporting Information). Samples were analyzed at predetermined intervals (30, 60, and 90 days). They were stored in aluminum foil pouches (10.3 cm × 17.7 cm) containing two desiccant canisters.^[^ ^28^ ^]^ To evaluate chemical stability, each patch was placed in a 10 mL volumetric flask and diluted with an 80/20 water/acetonitrile solution. The mixture was stirred for 1 hour until the solution was clear, then transferred to a vial for HPLC analysis. Chemical stability was determined using a reversed‐phase Waters HClass system with a UV detector. Separation was performed on an XBridge C18 column (3.5 µm particle size, 4.6 mm internal diameter, 150 mm length). The elution was carried out using a mobile phase of 5 mm ammonium phosphate, pH 3, and acetonitrile (as detailed in Table S6, Supporting Information) at a flow rate of 1 mL min^−1^. The analysis was conducted at 30°C with a 5 µL injection volume over 30 minutes. For physical stability, n = 2 MAPs were carefully scraped off the patch backing using a glass microscope slide. The MAPs were evenly spread on a zero‐background shallow holder and analyzed using PXRD from 2 to 40°2θ. PXRD data were collected on a Bruker D8 Advance system with Bragg‐Brentano geometry in a 35‐minute acquisition. ex vivo skin penetration and mechanical testing were carried out on n = 3 needles for each condition.

### Rodent Pharmacokinetic and Delivery Efficiency Studies

Pharmacokinetic studies on rats following intramuscular injection of an islatravir suspension were conducted at Merck & Co., Inc., West Point, Pennsylvania, USA, under an IACUC‐approved protocol (obtained from Merck & Co., Inc., West Point, PA, USA). The studies were performed in a climate‐controlled environment with temperatures ranging from 20 to 23°C and relative humidity between 44% and 57%. Islatravir microparticles were suspended in a vehicle containing sodium carboxymethylcellulose, polysorbate 80, and sucrose, and administered via a 70 µL injection at a concentration of 90 mg mL^−1^ into the quadriceps muscle. Approval for microneedle rodent pharmacokinetic studies was obtained from the Biological Services Unit Committee at Queen's University Belfast, with all researchers holding personal licenses from the UK Home Office. Female Sprague–Dawley rats (Charles River Laboratories, Harlow, UK), aged 8–10 weeks, were acclimatized for a week before the experiments. For the MAP‐treated cohort, dorsal hair was removed 24 hours before the study using clippers (Remington Co., London, UK) and depilatory cream (Boots Smooth Care, Boots, Nottingham, UK) under 2–4% isoflurane anesthesia. Rats were left to recover for 24 hours to ensure their skin's barrier function was fully restored before MAP application. The following day, MAPs were applied using firm thumb pressure, with MAPs secured in Microfoam adhesive frames, covered with Tegaderm film, and further fastened with kinesiology tape to ensure MAPs remained in place for 24 hours. Each rat received four MAPs from Formulation F1, which were removed after 24 hours. In one study, blood plasma concentrations were monitored over 3 months, with samples collected via tail vein bleeds in 1.5 mL EDTA tubes, centrifuged at 2200 × *g* for 10 minutes at 4°C to separate plasma, and stored at −20°C for later analysis. In another study, four MAPs were applied to six rats for 24 hours, with blood plasma collected at 1, 5, 24, 29, and 48 hours before sacrificing the animals and resecting the skin at the application site. To determine systemic drug release, plasma concentrations were deconvoluted against intravenous islatravir data using Phoenix 64 WinNonLin software. A fit‐for‐purpose method was used to quantify islatravir in plasma and skin tissues. Plasma analysis involved a 0.025 mL aliquot with a quantitation range of 0.030 to 40 ng mL^−1^. Samples were fortified with stable isotope‐labeled standards and extracted with 1 m ammonium acetate (pH 5) and acetonitrile, then analyzed using reverse‐phase chromatography with water (0.2 mm ammonium fluoride) and methanol, and detected using a Sciex API 6500+ triple quadrupole mass spectrometer in MRM mode. For skin tissue analysis, the entire sample was weighed and homogenized in 70/30 methanol‐water, using stainless steel beads in a Geno/Grinder (Cole‐Parmer, Metuchen, NJ, USA). The homogenate was fortified with internal standards, precipitated with acetonitrile, and analyzed under the same LC–MS/MS conditions as the plasma samples.

### Fabrication and Characterization of Larger MAPs

Larger MAPs were fabricated using a similar technique as previously described. Briefly, F1 and F2 (outlined in Table S3, Supporting Information) were selected to be cast into larger MAP molds (1110 bilayer pyramidal/cuboidal tips per patch, 850 µm height, 300 µm base width, 100 µm interspacing and 1.76 cm^2^ patch area). Following casting tip and baseplate layers, the formed larger MAP patches were dried further at 37°C for 24 hours and later subjected to several characterization, namely height reduction, skin insertion, drug content, and ex vivo skin deposition study, with methods equivalent to those described for the smaller patches above.

### Minipig PK Studies: Study Design and Bioanalytical Methods

Approval for minipig pharmacokinetic studies and animal handling and welfare was performed in accordance with Labcorp Laboratories Limited standard operating procedures. In order to determine the drug delivery efficiency and pharmacokinetics from administration of islatravir MAPs, 16 male naïve 13 to 14 week old Gottingen minipigs weighing between 7 and 11 kg were obtained from Ellegaard Gottingen Minipigs A/S. On receipt, all animals were examined for external signs of ill health. The animals were acclimatized for a minimum of 4 weeks, during which time their health status was monitored. A veterinary inspection was performed before the start of dosing to ensure suitability for the study. The 16 minipigs were divided into four different dose groups as shown in Table S6 (Supporting Information). Techniques for application of MAPs were equivalent to those described for rodents, above, with removal of patches after a 24 hours wear time for group C and after a 48 hours wear time for group D. Ten large MAPs were applied to each minipig, with each patch applied to the abdominal skin along the ribcage. Blood samples measuring 0.5 mL in volume were collected pre‐dose and then at 5, 10, 15, 30 minutes, 1, 2, 4, 7, 24, 48 hours following dosing for group A and at 2, 4, 24, 48, 72, 96, 120 h, then Days 13, 20, 27, 34, 41, 48, 55, 62, 69, 76, 83, 90 following dosing for groups B through D. Blood was collected into blood tubes containing K_2_EDTA anticoagulant and centrifuged (1500 × *g*, 10 min, 4°C) to prepare plasma for analysis. Skin samples (dose site area) were excised from Group C and D animals at necropsy. LC‐MS/MS analysis was employed to determine the concentrations of minipig plasma and skin. For plasma, a 0.05 mL aliquot was used, establishing a lower limit of quantitation of 0.030 ng mL^−1^ and a dynamic range from 0.030 to 40 ng mL^−1^. Samples underwent fortification with stable‐isotope labeled internal standards and were processed via salt‐assisted liquid‐liquid extraction using 1 m ammonium acetate (pH 5) and acetonitrile. Processed samples were chromatographed on a Waters (Milford, MA, USA) HSS T3 C18 column (50×3.0 mm, 2.5 µm) employing gradient elution with a mixture of water and methanol containing 0.1% propionic acid. Detection was carried out on a Sciex API 6500 triple quadrupole mass spectrometer, monitoring precursor‐to‐product ion combinations in MRM mode. Skin tissue analysis involved a homogenization process. The skin sample's entire weight was transferred to a 15 mL polypropylene centrifuge tube, followed by the addition of 19× volume of 70/30 methanol‐water (assuming a density of 1 g mL^−1^, resulting in a 20x dilution). Stainless steel beads were included, and the tube was capped and sealed with Parafilm. The tubes were homogenized using a SPEX (Cole‐Parmer, Metuchen, NJ) Geno/Grinder for two cycles of 5 minutes at maximum rpm (1750 rpm). Using a 0.01 mL aliquot, the lower limit of quantitation for skin samples was determined to be 1.13 ng mL^−1^, with a dynamic range from 1.13 to 1500 ng mL^−1^. After fortifying sample aliquots with stable labelled internal standards, homogenate samples were precipitated with acetonitrile before injection. LC–MS/MS conditions matched those used for minipig plasma analysis.

### Modelling Absorption and Human Dose Projections

In order to determine the kinetics of drug absorption, drug plasma concentration after IM/MAP administration was deconvoluted against intravenous pharmacokinetic in Phoenix 64 WinNonLin (Build 8.1.0. 3530, Certara, Princeton, New Jersey). Human pharmacokinetic was modeled by convoluting the dose‐normalized input rate (calculated by dividing input rate in mg d^−1^ from the deconvolution by the dose in mg) with modeled human intravenous pharmacokinetic, with exponential terms derived from fitting the oral PK from a single dose at 1, 2, 10 and 30 mg and multiple doses at 0.75 mg assuming 100% bioavailability. Dose‐normalized human PK projections were then multiplied by a 322 mg dose to calculate projected plasma concentrations after dosing MAPs with a total surface area of 20 cm^2^.

### Statistical analysis

Statistical analysis of the data was carried out utilizing GraphPad Prism version 8.0 (GraphPad Software, San Diego, California, USA). One‐way analysis of variance (ANOVA) was utilized to compare multiple groups across various aspects. A significance threshold of *p* < 0.05 was set for all statistical analyses.

## Conflict of Interest

Ashley R. Johnson, Steven Tignor, Jeanine Ballard, Nathan Rudd, and Angela Wagner are employees of employees of Merck Sharp & Dohme LLC, a subsidiary of Merck & Co., Inc., Rahway, NJ, USA. Merck Sharp & Dohme LLC, a subsidiary of Merck & Co., Inc., Rahway, NJ, USA has provided funding for this work.

## Author Contributions

**Qonita Kurnia Anjani**: Conceptualization, Methodology, Visualization, Investigation, Validation, Formal analysis, Data curation, Writing – original draft, Writing – review & editing. **Ashley R. Johnson**: Conceptualization, Methodology, Formal analysis, Funding Acquisition, Project Administration, Resources, Visualization, Supervision, Writing – original draft, Writing – review & editing. **Akmal H. Sabri**: Investigation, Formal analysis, Writing – original draft. **Ryan Lutz**: Methodology, Investigation, Validation, Formal analysis, Data curation, Writing – review & editing. **Steven Tignor**: Methodology, Investigation, Validation, Formal analysis, Data curation. **Jeanine Ballard**: Methodology, Investigation, Validation, Formal analysis, Data curation. **Nathan Rudd**: Methodology, Investigation, Validation, Formal analysis, Data curation. **Li Zhao**: Investigation. **Lalitkumar K. Vora**: Investigation. **Angela Wagner**: Funding Acquisition, Project Administration, Resources, Supervision. **Stephanie E. Barrett**: Funding Acquisition, Project Administration, Resources, Supervision. **Ryan F. Donnelly**: Funding Acquisition, Project Administration, Resources, Supervision, Writing – review & editing.

## Supporting information



Supporting Information

## Data Availability

The data that support the findings of this study are available from the corresponding author upon reasonable request.
