# Supplementary material for: Delivery of Islatravir via High Drug‐Load, Long‐acting Microarray Patches for the Prevention or Treatment of Human Immunodeficiency Virus
Source: Adv Healthc Mater. 2025 Jan 22;14(7):2403615. doi: 10.1002/adhm.202403615 (PMC11912095; doi:10.1002/adhm.202403615)
Supplement: Supplementary file 1 — Supporting Information [file ADHM-14-0-s001.pdf]

ADVANCED HEALTHCARE MATERIALS

Supporting Information

for *Adv. Healthcare Mater.*, DOI 10.1002/adhm.202403615

Delivery of Islatravir via High Drug-Load, Long-acting Microarray Patches for the Prevention or Treatment of Human Immunodeficiency Virus

Qonita Kurnia Anjani, Ashley R. Johnson, Akmal H. Sabri, Ryan Lutz, Steven Tignor, Jeanine Ballard, Nathan Rudd, Li Zhao, Lalitkumar K. Vora, Stephanie E. Barrett, Angela Wagner and Ryan F. Donnelly**

Supporting Information

Delivery of Islatravir *via* High Drug-Load, Long-acting Microarray Patches for the Prevention or Treatment of Human Immunodeficiency Virus

Qonita Kurnia Anjani[1†], Ashley R. Johnson[2†], Akmal H. Sabri[1†], Ryan Lutz[3], Steven Tignor[4], Jeanine Ballard[3], Nathan Rudd[5], Li Zhao[1], Lalitkumar K. Vora[1], Angela Wagner[2], and Ryan F. Donnelly[1]**

[†]Authors contributed equally to this work

*Corresponding authors: Ashley R. Johnson (ashley.johnson@merck.com) and Ryan F. Donnelly (r.donnelly@qub.ac.uk)

1. School of Pharmacy, Queen's University Belfast, Medical Biology Centre, 97 Lisburn Road, Belfast BT9 7BL, UK.
2. Sterile and Specialty Products, Merck & Co., Inc., 2000 Galloping Hill Road, Kenilworth NJ 07033, USA.
3. Absorption, Distribution, Metabolism & Excretion, Merck & Co., Inc., 770 Sumneytown Pike, West Point, PA 19486, USA.
4. Small Molecule Analytical Research and Development, Merck & Co., Inc., Rahway NJ 07065, USA.
5. Pharmaceutical Sciences R&D, Merck Animal Health, 126 E. Lincoln Avenue, Rahway NJ 07065, USA.

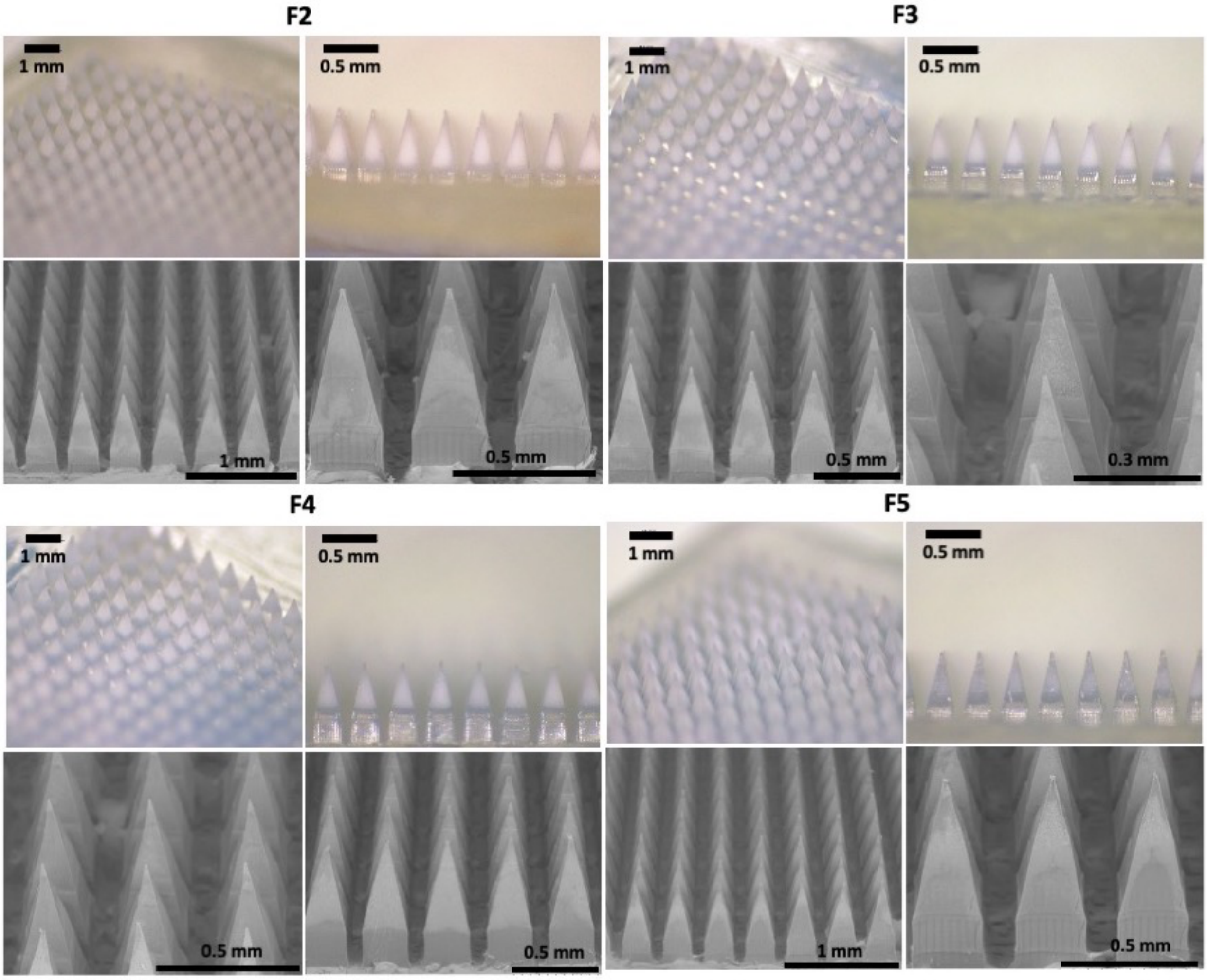

Figure S1. Digital and SEM images of islatravir loaded MAPs: F1, F2, F3, F4, and F5.

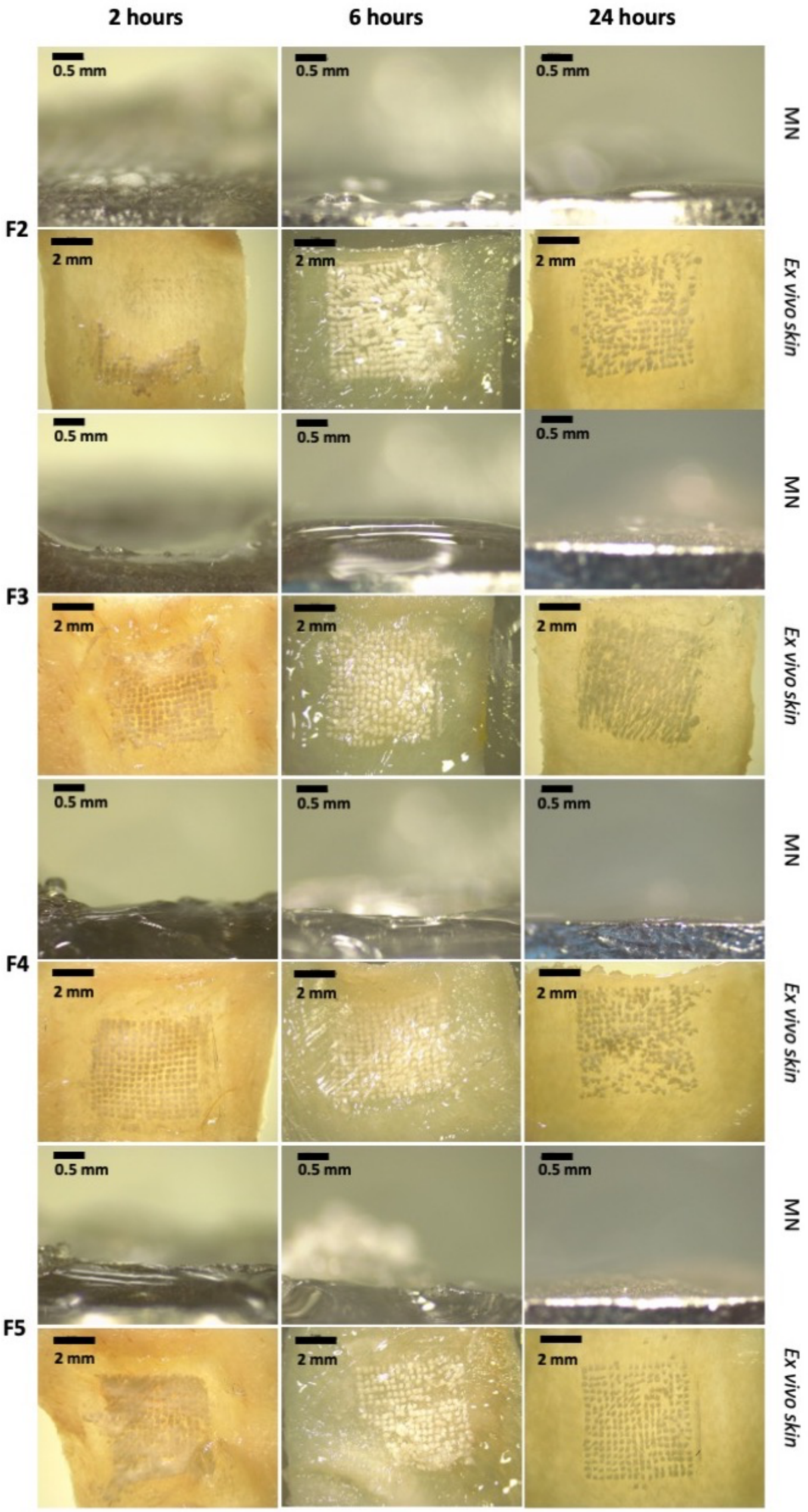

Figure S2. Images of respective islatravir loaded MAPs dissolving in the *ex vivo* neonatal porcine skin at 2, 6, and 24 hours acquired by optical microscopy.

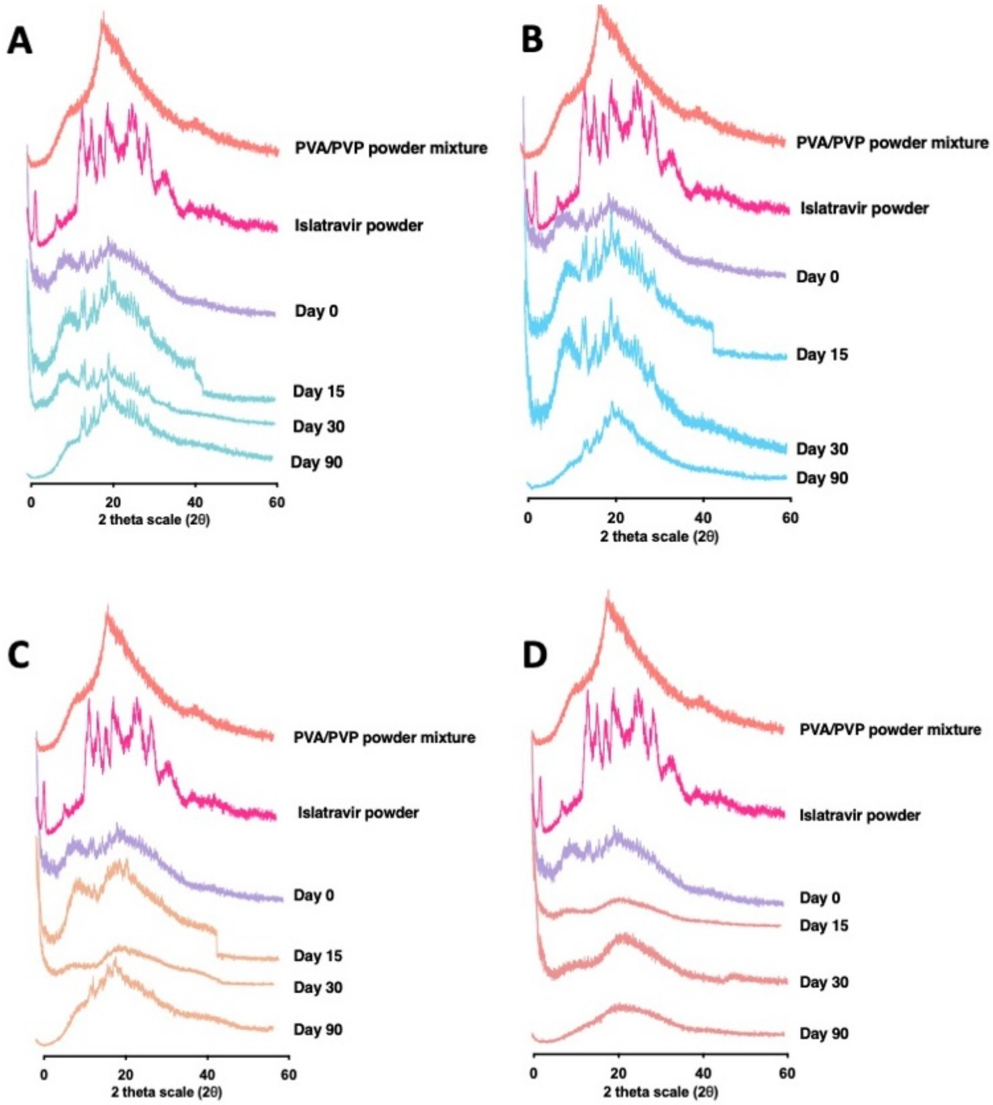

Figure S3. XRD diffractograms of islatravir-loaded dissolving MAPs stored under varying conditions: (A) 25°C/65% RH (in pouch), (B) 25°C/65% RH (out of pouch), (C) 40°C/75% RH (in pouch), and (D) 40°C/75% RH (out of pouch). Evaluations were conducted at different time points: day 0, day 15, day 30, and day 90.

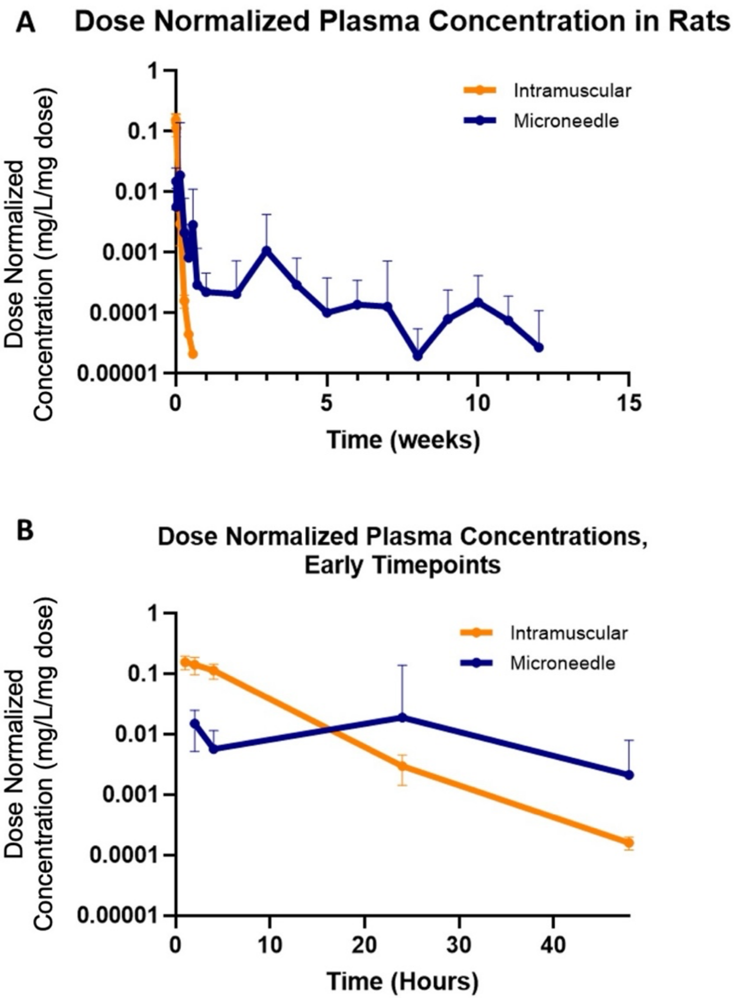

Figure S4. A) Dose normalized plasma concentrations in rats after administration of MAPs and an intramuscular suspension. B) Dose normalized plasma concentrations at early timepoints.

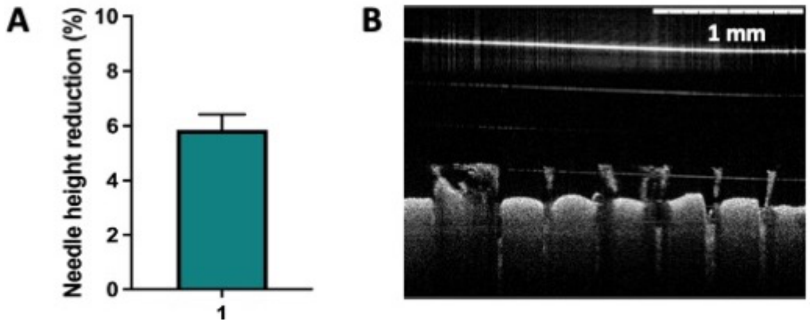

Figure S5. A) Assessment of microneedle height reduction after 32 N of force was applied to a large MAP. B) OCT imaging of a large MAP inside porcine tissue *ex vivo*

Table S1. Formulation three-month chemical stability at tested conditions (% LCAP).

	CMPD 1	CMPD 2	CMPD 3	CMPD 4	Islatravir	CMPD 5	CMPD 6
**t=0 Average**	0.08	1.33	0.06	0.32	97.93	0.09	0.21
**1M 5C/Closed**	0.08	1.32	0.06	0.26	98.03	0.08	0.18
**1M 30/65 Open**	0.09	1.33	0.06	0.26	98.00	0.08	0.18
**1M 40/75 Closed**	0.08	1.33	0.06	0.26	98.03	0.08	0.18
**1M 40/75 Open**	0.08	1.34	0.06	0.26	97.99	0.08	0.18
**3M 40/75 Open**	0.05	1.41	0.06	0.27	97.95	0.08	0.19

Table S2. Storage condition of MAPs in stability study.

Code	Storage condition	In/out pouch
Condition 1	2-8°C/Ambient humidity	In pouch
Condition 2	25°C/65% RH	In pouch
Condition 3	25°C/65% RH	Out of pouch
Condition 4	40°C/75% RH	In pouch
Condition 5	40°C/75% RH	Out of pouch

Table S3. Formulation of MAP tips.

Component (w/w)	F1	F2	F3	F4	F5
Islatravir	39.5%	30.0%	25.0%	20.0%	15.0%
PVA 9-10 kDa	2.8%	3.0%	2.5%	2.0%	1.5%
PVP 58 kDa	2.8%	3.0%	2.5%	2.0%	1.5%
Deionised water	54.9%	64.0%	70.0%	76.0%	82.0%

Table S4. Formulation of MAP baseplate layer.

Component	Composition (w/w)
PVP 1,300 kDa	30.0%
Glycerol	1.5%
DI water	68.5%

Table S5. Composition of mobile phase for quantification of islatravir in the *in vitro* studies.

Time (min)	0.1% v/v phosphoric acid (%)	Acetonitrile (%)
0.00	80	20
3.00	65	35
6.00	10	90
6.01	80	20
10.00	80	20

Table S6. Composition of mobile phase for chemical stability determination of islatravir in the *in vitro* studies.

Time (min)	5 mM Ammonium Phosphate, pH 3 (%)	Acetonitrile (%)
0.00	95	5
1.00	95	5
8.00	90	10
18.00	65	35
25.00	20	80
25.01	95	5
30.00	95	5

Table S7. Minipig study design

Dose group	Route of administration	Formulation	Application time	Dose level	N#
**A**	Intravenous	Solution in 20% Captisol	Bolus	5 mg/kg	4
**B**	Intramuscular	Aqueous suspension	Bolus	2 mg/kg	4
**C**	MAP	Formulation F1	24 hour	255 mg/animal	4
**D**	MAP	Formulation F1	48 hours	255 mg/animal	4
